# Respiratory Syncytial Virus Prefusion F Protein Vaccine Is Efficacious in Older Adults With Underlying Medical Conditions

**DOI:** 10.1093/cid/ciad471

**Published:** 2023-09-12

**Authors:** Robert G Feldman, Raffaele Antonelli-Incalzi, Katie Steenackers, Dong-Gun Lee, Alberto Papi, Michael G Ison, Laurence Fissette, Marie-Pierre David, Céline Maréchal, Marie Van der Wielen, Lusine Kostanyan, Veronica Hulstrøm, for the AReSVi-006 Study Group, Mark Adams, Michael Adams, Elaine Jacqueline Akite, Ingrid Alt, Charles Andrews, Asmik Asatryan, Eugene Athan, Ghazaleh Bahrami, Elena Bargagli, Qasim Bhorat, Paul Bird, Przemyslaw Borowy, Celine Boutry, Carles Brotons Cuixart, David Browder, Judith Brown, Erik Buntinx, Donald Cameron, Laura Campora, Kenneth Chinsky, Melissa Choi, Eun-Ju Choo, Delphine Collete, Maria Corral Carrillo, Matthew G Davis, Magali de Heusch, Ferdinandus de Looze, Marc De Meulemeester, Ferdinando De Negri, Nathalie De Schrevel, David DeAtkine, Viktoriya Dedkova, Dominique Descamps, Nancy Dezutter, Peter Dzongowski, Tamara Eckermann, Brandon Essink, Karen Faulkner, Murdo Ferguson, Gregory Fuller, Isabel Maria Galan Melendez, Ivan Gentile, Wayne Ghesquiere, Doria Grimard, Olivier Gruselle, Scott Halperin, Amardeep Heer, Laura Helman, Andre Hotermans, Tomas Jelinek, Jackie Kamerbeek, Hyo Youl Kim, Murray Kimmel, Mark Koch, Satu Kokko, Susanna Koski, Shady Kotb, Antonio Lalueza, Joanne M Langley, Jin-Soo Lee, Isabel Leroux-Roels, Muriel Lins, Johannes Lombaard, Akbar Mahomed, Mario Malerba, Celine Marechal, Federico Martinon-Torres, Jean-Benoit Martinot, Cristina Masuet-Aumatell, Damien McNally, Carlos Eduardo Medina Pech, Jorge Mendez Galvan, Narcisa Elena Mesaros, Dieter Mesotten, Essack Mitha, Kathryn Mngadi, Beate Moeckesch, Barnaby Montgomery, Linda Murray, Rhiannon Nally, Silvia Narejos Perez, Joseph Newberg, Paul Nugent, Dolores Ochoa Mazarro, Harunori Oda, Aurelie Olivier, Maurizio Orso, Jacinto Ortiz Molina, Tatiana Pak, Dae Won Park, Meenakshi Patel, Minesh Patel, Anna Maria Pedro Pijoan, Merce Perez Vera, Alberto Borobia Perez, Lina Perez-Breva, Claudia Pileggi, Fabrizio Pregliasco, Carol Pretswell, Dean Quinn, Michele Reynolds, Viktor Romanenko, Jeffrey Rosen, Nathalie Roy, Belen Ruiz Antoran, Hideaki Sakata, Joachim Sauter, Axel Schaefer, Tino F Schwarz, Izabela Sein Anand, Jose Antonio Serra Rexach, David Shu, Andres Siig, William Simon, Svetlana Smakotina, Brigitte Stephan, Silvio Tafuri, Kenji Takazawa, Guy Tellier, Wim Terryn, Leslie Tharenos, Nick Thomas, Nicole Toursarkissian, Benita Ukkonen, Noah Vale, Pieter-Jan Van Landegem, Richard N van Zyl-Smit, Carline Vanden Abeele, Celine Verheust, Lode Vermeersch, Miguel Vicco, Francesco Vitale, Olga Voloshyna, Judith White, Seong-Heon Wie, Jonathan Wilson, Pedro Ylisastigui

**Affiliations:** Senior Clinical Trials Inc., Laguna Hills, California, USA; Department of Internal Medicine and Geriatrics, Università Campus Bio-Medico di Roma, Rome, Italy; Centre for the Evaluation of Vaccination, University of Antwerp, Antwerp, Belgium; Division of Infectious Diseases, Department of Internal Medicine, Seoul St. Mary's Hospital, The Catholic University of Korea, Seoul, South Korea; Pulmonary Division, University of Ferrara, St. Anna University Hospital, Ferrara, Italy; Respiratory Diseases Branch, Division of Microbiology and Infectious Diseases, National Institute of Allergy and Infectious Diseases, National Institutes of Health, Rockville, Maryland, USA; Vaccines R&D, GSK, Wavre, Belgium; Vaccines R&D, GSK, Wavre, Belgium; Vaccines R&D, GSK, Wavre, Belgium; Vaccines R&D, GSK, Wavre, Belgium; Vaccines R&D, GSK, Wavre, Belgium; Vaccines R&D, GSK, Wavre, Belgium

**Keywords:** cardiorespiratory, comorbidity, respiratory tract illness, RSV, vaccination

## Abstract

**Background:**

Older adults with chronic cardiorespiratory or endocrine/metabolic conditions are at increased risk of respiratory syncytial virus (RSV)-related acute respiratory illness (RSV-ARI) and severe respiratory disease. In an ongoing, randomized, placebo-controlled, multicountry, phase 3 trial in ≥60-year-old participants, an AS01_E_-adjuvanted RSV prefusion F protein-based vaccine (RSVPreF3 OA) was efficacious against RSV-related lower respiratory tract disease (RSV-LRTD), severe RSV-LRTD, and RSV-ARI. We evaluated efficacy and immunogenicity among participants with coexisting cardiorespiratory or endocrine/metabolic conditions that increase the risk of severe RSV disease (“conditions of interest”).

**Methods:**

Medically stable ≥60-year-old participants received 1 dose of RSVPreF3 OA or placebo. Efficacy against first RSV-LRTD and RSV-ARI episodes was assessed in subgroups with/without coexisting cardiorespiratory or endocrine/metabolic conditions of interest. Immunogenicity was analyzed post hoc in these subgroups.

**Results:**

In total, 12 467 participants received RSVPreF3 OA and 12 499 received placebo. Of these, 39.6% (RSVPreF3 OA) and 38.9% (placebo) had ≥1 coexisting condition of interest. The median efficacy follow-up was 6.7 months. Efficacy against RSV-LRTD was high in participants with ≥1 condition of interest (94.6%), ≥1 cardiorespiratory (92.1%), ≥1 endocrine/metabolic (100%), and ≥2 conditions of interest (92.0%). Efficacy against RSV-ARI was 81.0% in participants with ≥1 condition of interest (88.1% for cardiorespiratory, 79.4% for endocrine/metabolic conditions) and 88.0% in participants with ≥2 conditions of interest. Postvaccination neutralizing titers were at least as high in participants with ≥1 condition of interest as in those without.

**Conclusions:**

RSVPreF3 OA was efficacious against RSV-LRTD and RSV-ARI in older adults with coexisting medical conditions associated with an increased risk of severe RSV disease.

**Clinical Trials Registration:**

ClinicalTrials.gov: NCT04886596.

## Graphical Abstract

**Graphical Abstract:**
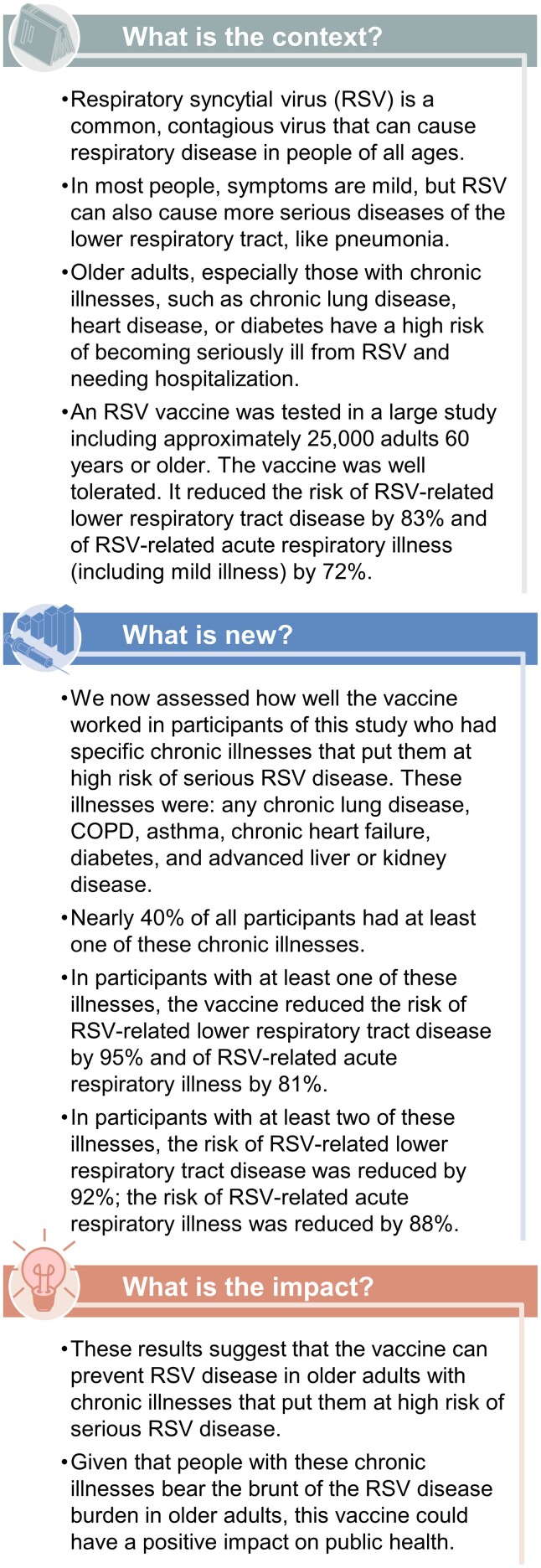

Respiratory syncytial virus (RSV) is an important cause of acute respiratory illness (ARI) in older adults, with clinical presentations ranging from mild, cold-like symptoms, to severe lower respiratory tract disease (LRTD) [1–5]. A meta-analysis based on studies reporting the incidence of medically attended RSV in adults in the United States estimated that each year, there are approximately 159 000 hospitalizations, 119 000 emergency department admissions, and 1.4 million outpatient visits from RSV among ≥65-year-old people in the United States [6]. In 2019, an estimated 5.2 million RSV-related ARI (RSV-ARI) cases, 470 000 RSV-related hospitalizations, and 33 000 RSV-related in-hospital deaths occurred in adults aged ≥60 years in high-income countries [5].

Older adults with underlying cardiorespiratory and endocrine or metabolic conditions (such as chronic obstructive pulmonary disease [COPD], congestive heart failure, and diabetes) are at increased risk of RSV-ARI [7, 8]. This population often has more severe clinical presentations that may require medical care and may lead to hospitalization, exacerbation of underlying conditions, or death [5, 7–19]. A prospective, population-based, surveillance study in New York state showed that the RSV incidence in hospitalized adults was approximately 15 times higher among ≥65-year-old people than among 18- to 49-year-old people. Moreover, the incidence in older hospitalized adults with COPD, coronary artery disease, congestive heart failure, and diabetes were 4 to 13 times, 4 to 6 times, 4 to 8 times, and 2 to 6 times higher, respectively, than in older adults without these conditions [10]. Older adults hospitalized with RSV also show a functional decline that may persist for several months after hospital discharge and, in some, may lead to loss of previous independence [20].

With supportive care remaining the current clinical standard for RSV treatment and considering the high prevalence of multimorbidity in older adults [21–23], there is a high unmet medical need for RSV vaccines in older adults that are also effective in those with chronic medical conditions. Several RSV vaccines are under development [24, 25], and an adjuvanted RSV vaccine for older adults (RSVPreF3 OA, *Arexvy*, GSK), based on the RSV fusion (F) protein stabilized in its prefusion conformation, was recently approved [26]. In the ongoing phase 3 AReSVi-006 trial, RSVPreF3 OA was immunogenic and showed 82.6% efficacy against RSV-related LRTD (RSV-LRTD), 94.1% against severe RSV-LRTD, and 71.7% against RSV-ARI in adults ≥60 years of age during 1 RSV season [27]. The vaccine had an acceptable safety profile [27]. The analyses reported here aimed to evaluate vaccine efficacy and immunogenicity of a single dose of RSVPreF3 OA in adults aged ≥60 years with coexisting cardiorespiratory and endocrine or metabolic conditions associated with an increased risk of severe RSV disease.

## METHODS

### Trial Design and Participants

The ongoing, randomized, placebo-controlled, phase 3 AReSVi-006 trial is being conducted in 17 countries in Africa, Asia, Australia, Europe, and North America. The primary objective was to demonstrate the efficacy of a single dose of RSVPreF3 OA in preventing RSV-LRTD during 1 RSV season among adults aged ≥60 years. Results for the primary and main secondary objectives and detailed methods have been reported previously [27]. Adults aged 60 years or older were enrolled after providing written or witnessed informed consent if the investigator considered that they would be able to comply with the protocol requirements. Persons were excluded if they had previously received an RSV vaccine. Persons with chronic medical conditions could be enrolled if the investigator considered the person medically stable. Vaccines other than the study vaccine were allowed if administered more than 30 days before or after RSVPreF3 OA or placebo administration (or more than 14 days for inactivated or subunit influenza and COVID-19 vaccines) [27].

Participants were randomized 1:1 (using an automated internet-based system) to receive 1 intramuscular dose of RSVPreF3 OA (containing 120 µg of RSVPreF3 antigen and the AS01_E_ adjuvant system) or placebo before the start of the RSV season. Personnel not involved in data collection or evaluation administered the injections. Participants and study team members responsible for evaluating study endpoints were blinded. Participant follow-up is planned for 3 RSV seasons in the Northern Hemisphere and at least 2 seasons in the Southern Hemisphere. Results for the current analyses are based on data available at the end of the first Northern Hemisphere RSV season (April 2022).

The trial is registered on ClinicalTrials.gov (NCT04886596). The relevant independent ethics committees or institutional review boards for each trial site approved the protocol and amendments (protocol summary available on https://www.gsk-studyregister.com/en/trial-details/?id=212494 and full protocol as an appendix to Papi et al [27]). The trial is conducted in accordance with Good Clinical Practice guidelines, the principles of the Declaration of Helsinki, and local laws and regulations. An independent data monitoring committee is monitoring the participants' safety.

### Efficacy Assessments

Surveillance for ARI was done through spontaneous reporting by the participants (starting on day 1 [i.e., day of vaccine/placebo administration]) and through scheduled site staff contacts with the participants (starting on day 31), as previously described [27]. Scheduled contacts (by phone, email, text message, or other means) occurred every 2 weeks during the RSV season and every month between seasons, with a season lasting from 1 October to 30 April in the Northern Hemisphere and from 1 March to 30 September in the Southern Hemisphere. For each suspected ARI, an assessment visit was planned (ideally 48 hours and maximum 6 days after ARI onset), during which the investigator or staff member performed a physical examination, measured body temperature, respiratory rate, and oxygen saturation to assess if the ARI met the case definition. If the ARI met the case definition, nasal and throat swabs were obtained by the site staff. Starting from day 31, participants also performed nasal self-swabs (preferably within 48 hours and maximum 5 days after ARI onset). Swabs were used to test for the presence of RSV-A and RSV-B subtypes by quantitative reverse transcriptase-polymerase chain reaction [27].

ARI was defined as the presence of at least 2 respiratory symptoms or signs or at least 1 respiratory and 1 systemic symptom or sign, lasting for at least 24 hours. LRTD was defined as the presence of at least 2 lower respiratory symptoms or signs (including at least 1 lower respiratory sign) or at least 3 lower respiratory symptoms, lasting for at least 24 hours (Supplementary methods). Upper respiratory symptoms were nasal congestion/rhinorrhea and sore throat; lower respiratory symptoms were new/increased sputum, cough, or dyspnea; lower respiratory signs were new/increased wheezing, or crackles/rhonchi, increased respiratory rate, low/decreased oxygen saturation, and need for oxygen supplementation; systemic symptoms/signs were fever/feverishness, fatigue, body aches, headache, and decreased appetite. All LRTD cases (meeting the case definition or reported by the investigator but not meeting the case definition) that were confirmed by quantitative reverse transcriptase-polymerase chain reaction to be RSV-associated were reviewed by an external adjudication committee (Supplementary methods). Only cases confirmed as RSV-LRTD by this committee were included in the current analyses [27].

### Immunogenicity Assessments

The humoral immune response was evaluated on serum samples collected on day 1 (prevaccination) and day 31 (1 month postvaccination) from a subset of participants in selected countries and sites (immunogenicity subset). RSV-A and RSV-B neutralizing titers were quantified using neutralization assays with assay cutoffs of 18 estimated dilution 60 (ED60) for RSV-A and 30 ED60 for RSV-B. RSVPreF3-binding immunoglobulin G concentrations were quantified using an enzyme-linked immunosorbent assay, with an assay cutoff of 25 enzyme-linked immunosorbent assay units/mL [27].

### Selection of Coexisting Medical Conditions of Interest

Subgroup analyses reported here were based on the presence or absence of selected coexisting medical conditions that are known to increase the risk of severe RSV disease [28], complemented with relevant risk factors of influenza complications [29], collectively referred to as “conditions of interest.” These included cardiorespiratory conditions (any chronic respiratory or pulmonary disease [including COPD, asthma, and other conditions] and chronic heart failure) and endocrine and metabolic conditions (diabetes mellitus type 1 or 2 and advanced liver or renal disease). Information about coexisting medical conditions was gathered by interviewing the participants and/or reviewing their medical records. To generate the subgroups, the selected medical conditions of interest were identified in the database using a predefined list of Medical Dictionary for Regulatory Activities Terminology preferred terms.

### Statistical Analyses

We planned to enroll up to 25 000 participants to be able to demonstrate the primary objective and to include 1800 participants in the immunogenicity subset, as described previously [27]. Vaccine efficacy analyses were performed on the modified exposed population, which included all participants who had received RSVPreF3 OA or placebo and did not report an RSV-ARI before day 15 postvaccination. Vaccine efficacy for first occurrence of RSV-ARI and RSV-LRTD was calculated as 1 minus the relative risk with the conditional exact binomial method based on a Poisson model. The period at risk started on day 15 postvaccination and ended at the first occurrence of an event or censoring. Vaccine efficacy was assessed in participants who (at baseline) reported none of the medical conditions of interest, at least 1 of these conditions, at least 1 of the cardiorespiratory conditions, or at least 1 of the endocrine and metabolic conditions of interest. In addition, efficacy was analyzed post hoc in participants with at least 2 conditions of interest. We also assessed vaccine efficacy against RSV-LRTD and RSV-ARI with medically attended visits (post hoc analysis, Supplementary results). Medically attended visits included visits with a general practitioner or specialist and emergency department visits.

Immunogenicity was analyzed in the per-protocol population for immunogenicity, which included all participants in the immunogenicity subset who had received RSVPreF3 OA or placebo, had postvaccination immunogenicity data available, and adhered to the protocol. Geometric mean titers (GMTs) and geometric mean concentrations (GMCs) were calculated with 95% confidence intervals (CIs) by taking the antilogarithm of the arithmetic mean of the log_10_-transformed titers or concentrations. Titers or concentrations below the assay cutoffs were given an arbitrary value of half the assay cutoffs; those above the assays' upper limits of quantification were given an arbitrary value of the upper limits of quantification. Geometric mean increases, defined as the geometric mean of the within-participant ratios of the postvaccination titer or concentration over the prevaccination titer or concentration, were calculated with 95% CIs. These immunogenicity variables were analyzed post hoc in the different medical condition of interest subgroups.

Missing data for efficacy and immunogenicity analyses were not replaced or imputed. All statistical analyses were done using SAS Life Science Analytics Framework.

## RESULTS

### Trial Population

**Figure 1.**
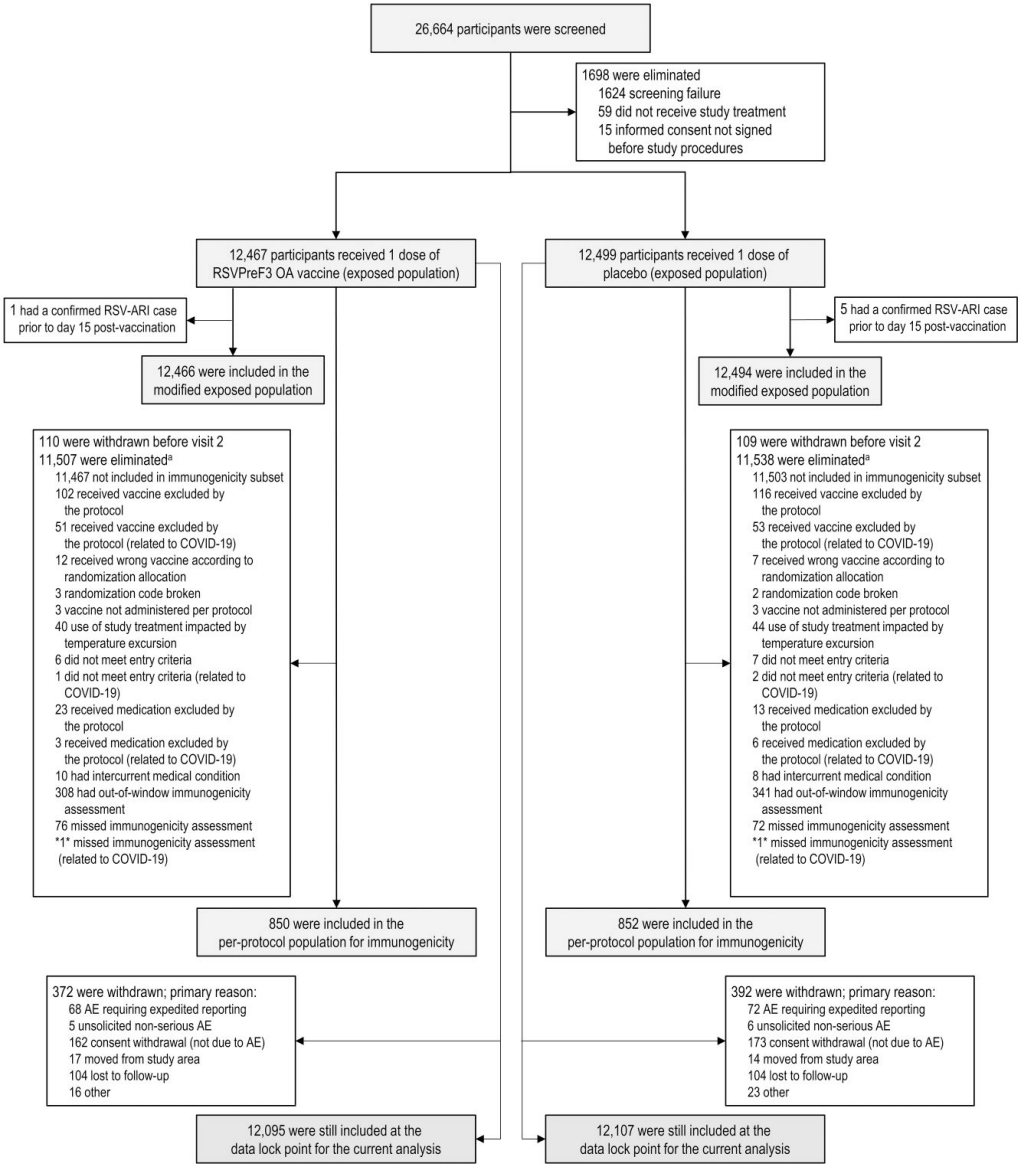
Participant flow diagram. Numbers between asterisks (eg, *1*) indicate that data by group are blinded to avoid participant-level unblinding of the study team; the number between the asterisks shows the total number across the 2 groups. AE, adverse event; RSV, respiratory syncytial virus; RSV-ARI, respiratory syncytial virus–related acute respiratory illness; RSVPreF3 OA, AS01_E_-adjuvanted respiratory syncytial virus prefusion F protein–based vaccine. ^a^More than 1 reason for elimination can occur for 1 participant.

Between May 2021 and January 2022, 26 664 adults aged ≥60 years old were enrolled in the trial, of whom 24 966 were part of the exposed population (12 467 received RSVPreF3 OA and 12 499 received placebo) (Figure 1). Of these, more than 95.0% of participants in both groups had at least 1 coexisting general medical condition (Table 1). The most common conditions were vascular hypertensive disorders (57.2% of all participants in the exposed population), osteoarthropathies (33.5%), elevated cholesterol (24.8%), and diabetes mellitus (22.9%) (Supplementary Table 1). In total, 39.6% (RSVPreF3 OA) and 38.9% (placebo) of participants had at least 1 of the coexisting medical conditions of interest (i.e., associated with severe RSV disease); 20.0% and 19.4% had at least 1 cardiorespiratory condition of interest, and 25.7% and 25.9% had at least 1 endocrine or metabolic condition of interest (mostly diabetes; Table 1). The mean body mass index in both groups was 29.1 kg/m^2^. Other baseline characteristics were also balanced between groups (Table 1 and shown previously [27]).

**Table 1.** Baseline Characteristics of the Trial Participants (Exposed Population)

Characteristic	RSVPreF3 OA N = 12 467	Placebo N = 12 499
Mean age (SD), y	69.5 (6.5)	69.6 (6.4)
Age group, n (%)		
60–69 y	6963 (55.9)	6980 (55.8)
70–79 y	4487 (36.0)	4491 (35.9)
≥80 y	1017 (8.2)	1028 (8.2)
Female sex, n (%)	6488 (52.0)	6427 (51.4)
Race, n (%)		
Asian	953 (7.6)	956 (7.6)
Black	1064 (8.5)	1101 (8.8)
White	9887 (79.3)	9932 (79.5)
Other	563 (4.5)	510 (4.1)
Mean BMI (SD), kg/m^2^	29.1 (6.1)	29.1 (6.0)
Any coexisting medical conditions, n (%)		
≥1 condition	11 929 (95.7)	11 905 (95.2)
Coexisting medical conditions of interest^a^, n (%)		
≥1 condition of interest	4937 (39.6)	4864 (38.9)
≥2 conditions of interest	2504 (20.1)	2434 (19.5)
≥3 conditions of interest	805 (6.5)	827 (6.6)
≥1 cardiorespiratory condition of interest	2496 (20.0)	2422 (19.4)
COPD	1131 (9.1)	1113 (8.9)
Asthma	1193 (9.6)	1113 (8.9)
Chronic respiratory/pulmonary disease	2223 (17.8)	2123 (17.0)
Chronic heart failure	398 (3.2)	403 (3.2)
≥1 endocrine and metabolic condition of interest	3200 (25.7)	3236 (25.9)
Diabetes mellitus type 1 or 2	2829 (22.7)	2877 (23.0)
Advanced liver or renal disease	667 (5.4)	676 (5.4)

Abbreviations: BMI, body mass index; COPD, chronic obstructive pulmonary disease; N, number of participants in the exposed population; n (%), number (percentage) of participants in the given category; placebo, group with participants who received a single dose of placebo; RSVPreF3 OA, group with participants who received a single dose of AS01E-adjuvanted respiratory syncytial virus (RSV) prefusion F protein-based vaccine; SD, standard deviation; y, years.

^a^Coexisting medical conditions of interest included cardiorespiratory conditions (any chronic respiratory or pulmonary disease [including COPD, asthma, and other conditions], chronic heart failure) and endocrine and metabolic conditions (diabetes mellitus type 1 or type 2 and advanced liver or renal disease) that are associated with an increased risk of severe RSV disease.

### Vaccine Efficacy Against RSV Disease in Participants With Coexisting Medical Conditions

A total of 24 960 participants were part of the modified exposed population for efficacy analyses. The median efficacy follow-up was 6.7 months. The RSV-LRTD incidence rates in the placebo group tended to be higher among participants with the selected coexisting conditions of interest (6.6–8.9/1000 person-years) than among those without any of these conditions (5.3/1000 person-years). A similar observation was made for the RSV-ARI incidence rates, which were 15.2–17.8/1000 person-years among placebo recipients with coexisting conditions of interest and 13.1/1000 person-years among those without these conditions (Table 2). Efficacy of RSVPreF3 OA against RSV-LRTD was 94.6% (95% CI, 65.9–99.9) in participants with at least 1 of the conditions of interest (92.1% [46.7–99.8] among those with cardiorespiratory conditions and 100% [74.0–100] among those with endocrine or metabolic conditions) and 92.0% (46.1–99.8) in participants with at least 2 of these conditions (Table 2).

**Table 2.** Vaccine Efficacy Against First Occurrence of RSV-LRTD and RSV-ARI (Modified Exposed Population)

	RSVPreF3 OA				Placebo				
Endpoint	N	n	T, p-y	n/T, n/1000 p-y	N	n	T, p-y	n/T, n/1000 p-y	Vaccine Efficacy, % (CI^a^)
RSV-LRTD									
RSV-LRTD, overall	12 466	7	6865.9	1.0	12 494	40	6857.3	5.8	82.6 (57.9–94.1)
RSV-LRTD by coexisting condition of interest^b^									
No condition of interest	7529	6	4094.1	1.5	7633	22	4148.1	5.3	72.5 (30.0–90.9)
≥1 condition of interest	4937	1	2771.8	0.4	4861	18	2709.1	6.6	94.6 (65.9–99.9)
≥1 cardiorespiratory condition of interest^c^	2496	1	1409.5	0.7	2421	12	1352.9	8.9	92.1 (46.7–99.8)
≥1 endocrine and metabolic condition of interest^d^	3200	0	1795.7	0.0	3234	13	1805.3	7.2	100 (74.0–100)
≥2 conditions of interest	2504	1	1418.2	0.7	2431	12	1362.8	8.8	92.0 (46.1–99.8)
RSV-ARI									
RSV-ARI, overall	12 466	27	6858.7	3.9	12 494	95	6837.8	13.9	71.7 (56.2–82.3)
RSV-ARI by coexisting condition of interest^b^									
No condition of interest	7529	19	4089.9	4.6	7633	54	4136.4	13.1	64.4 (39.0–80.1)
≥1 condition of interest	4937	8	2768.8	2.9	4861	41	2701.4	15.2	81.0 (58.9–92.3)
≥1 cardiorespiratory condition of interest^c^	2496	3	1408.5	2.1	2421	24	1349.0	17.8	88.1 (60.9–97.7)
≥1 endocrine and metabolic condition of interest^d^	3200	6	1793.2	3.3	3234	29	1800.0	16.1	79.4 (49.4–93.0)
≥2 conditions of interest	2504	3	1417.3	2.1	2431	24	1358.8	17.7	88.0 (60.5–97.7)

Cases of RSV-related LRTD and RSV-related ARI were reported up to the efficacy database lock of 11 April 2022. Cases were confirmed as positive for RSV-A or RSV-B subtypes by quantitative reverse transcriptase-polymerase chain reaction. RSV-LRTD cases were those identified by the adjudication committee. Vaccine efficacy was estimated using the Poisson method, with adjustment for age and geographic region.

Abbreviations: ARI, acute respiratory illness; CI, confidence interval; LRTD, lower respiratory tract disease; N, number of participants in the modified exposed population in the specified subgroup; n, number of participants with at least one RSV-LRTD or RSV-ARI; n/T, incidence rate of participants reporting at least 1 event; p-y, person-years; RSV, respiratory syncytial virus; RSVPreF3 OA, group with participants who received a single dose of AS01_E_-adjuvanted RSV prefusion F protein–based vaccine; placebo, group with participants who received a single dose of placebo; T, sum of follow-up time (from day 15 postvaccination until first occurrence of the event, data lock point, or dropout).

^a^96.95% CI for primary endpoint (RSV-LRTD, overall); 95% CI for other endpoints (no adjustment for multiplicity).

^b^Coexisting medical conditions of interest included cardiorespiratory conditions (chronic obstructive pulmonary disease [COPD], asthma, any chronic respiratory or pulmonary disease [including COPD, asthma, and other conditions], chronic heart failure) and endocrine and metabolic conditions (diabetes mellitus type 1 or type 2 and advanced liver or renal disease) that are associated with an increased risk of severe RSV disease.

^c^Of the 13 RSV-LRTD and 27 RSV-ARI cases among participants with cardiorespiratory conditions of interest, 13 RSV-LRTD and 24 RSV-ARI cases were among participants with chronic respiratory or pulmonary disease; 2 RSV-LRTD and 5 RSV-ARI cases were among participants with chronic heart failure.

^d^Of the 13 RSV-LRTD and 35 RSV-ARI cases among participants with endocrine and metabolic conditions of interest, 12 RSV-LRTD and 34 RSV-ARI cases were among participants with diabetes mellitus.

Efficacy of RSVPreF3 OA against RSV-ARI was 81.0% (95% CI, 58.9–92.3) in participants with at least 1 of the medical conditions of interest (88.1% [60.9–97.7] among those with cardiorespiratory conditions and 79.4% [49.4–93.0] among those with endocrine or metabolic conditions) and 88.0% (60.5–97.7) in participants with at least 2 of these conditions (Table 2).

### Immunogenicity in Participants With Coexisting Medical Conditions

**Figure 2.**
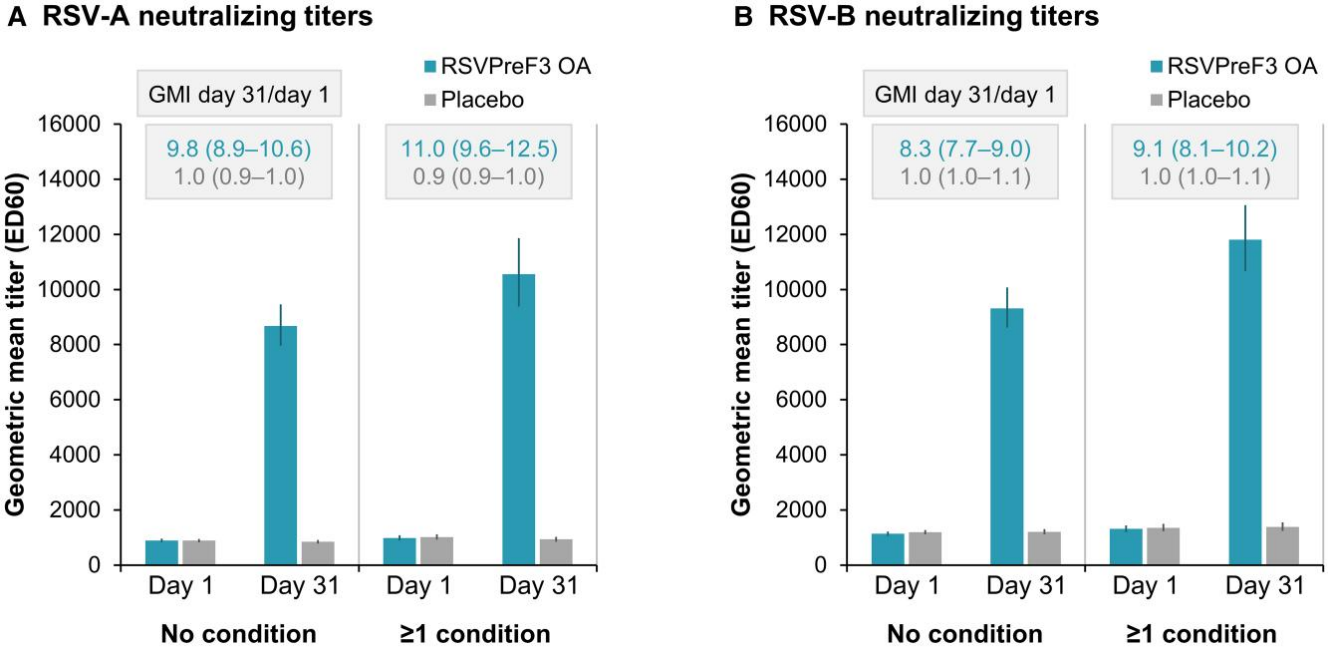
RSV-A and RSV-B neutralizing titers before and 1 month after RSVPreF3 OA or placebo administration, by coexisting medical conditions of interest (per-protocol population for immunogenicity). Graphs show respiratory syncytial virus (RSV) subtypes *A* and *B* neutralizing titers before (day 1) and 1 month after (day 31) administration of the AS01_E_-adjuvanted RSV prefusion F protein–based vaccine (RSVPreF3 OA) or placebo for the subgroups of participants without any of the coexisting medical conditions of interest (no condition) or with at least 1 of these conditions (≥1 condition); conditions of interest were cardiorespiratory conditions (chronic obstructive pulmonary disease [COPD], asthma, any chronic respiratory or pulmonary disease [including COPD, asthma, and other conditions], chronic heart failure) and endocrine and metabolic conditions (diabetes mellitus type 1 or type 2 and advanced liver or renal disease) that are associated with an increased risk of severe RSV disease. Error bars depict 95% confidence intervals. Rectangles above the bars contain geometric mean increases (GMIs) from day 1 to day 31 with 95% confidence intervals. ED60, estimated dilution 60.

Among participants with at least 1 of the coexisting conditions of interest, RSV-A– and RSV-B–neutralizing GMTs after a single dose of RSVPreF3 OA tended to be higher than GMTs among participants without any of these conditions (Figure 2, Supplementary Table 2). Geometric mean increases in neutralizing titers between day 1 (prevaccination) and day 31 after RSVPreF3 OA vaccination were in a similar range in the 2 subgroups: titers increased 11.0-fold for RSV-A and 9.1-fold for RSV-B in participants with at least 1 condition of interest compared with 9.8-fold (RSV-A) and 8.3-fold (RSV-B) in participants without these conditions (Figure 2, Supplementary Table 2). A similar profile was observed for RSVPreF3-binding immunoglobulin G responses, with a trend for higher postvaccination GMCs in participants with at least 1 medical condition of interest, and fold-increases of 13.3 in participants with and 12.9 in participants without any of these conditions (Supplementary Table 2).

## DISCUSSION

This large, randomized, placebo-controlled, multicountry, phase 3 trial enrolled a diverse population of adults aged ≥60 years, with age-related medical conditions representative of those occurring in the general older adult population [22, 30, 31]. More than 95% of participants had at least 1 coexisting general medical condition, and nearly 40% had at least 1 of the selected coexisting cardiorespiratory or endocrine and metabolic conditions associated with an increased risk of severe RSV disease. The efficacy of RSVPreF3 OA in preventing RSV-LRTD and RSV-ARI during 1 RSV season remained high in participants with at least 1 of these conditions of interest (94.6% for RSV-LRTD, 81.0% for RSV-ARI), at least 1 cardiorespiratory condition (92.1% for RSV-LRTD, 88.1% for RSV-ARI), at least 1 endocrine or metabolic condition (100% for RSV-LRTD, 79.4% for RSV-ARI), and at least 2 conditions of interest (92.0% for RSV-LRTD, 88.0% for RSV-ARI). Consistent with the high efficacy, RSVPreF3 OA elicited humoral immune responses in older adults with these medical conditions that were at least as high as immune responses in those without these conditions.

Older adults with chronic medical conditions have been shown to have a higher risk of symptomatic RSV-ARI, of requiring medically attended visits, and of progressing to severe disease [6–18, 32]. In our trial, we indeed observed higher background incidence rates of both RSV-ARI (up to 36% higher) and RSV-LRTD (up to 68% higher) among participants with 1 or more of the cardiorespiratory or endocrine and metabolic conditions of interest compared with those without any of these conditions. Considering the high efficacy of RSVPreF3 OA in older adults with coexisting medical conditions of interest observed in our trial, and the disproportionate contribution to the total RSV burden borne by this population [33], RSVPreF3 OA vaccination of this at-risk group could have a substantial impact on public health. The high efficacy against RSV-LRTD (87.5%) and RSV-ARI (79.0%) episodes with medically attended visits in our trial (Supplementary results) suggests that the vaccine could reduce the number of RSV-related medical visits and lower the burden on healthcare systems.

The observed neutralizing GMTs tended to be higher among participants with at least 1 coexisting condition of interest than among those without such conditions. In the absence of a correlate of protection, the clinical relevance of this finding is unknown. Nevertheless, efficacy point estimates also tended to be higher among those with these conditions versus those without, which could be due to more severe presentations of RSV-LRTD among those with the selected coexisting conditions.

Analyses on the total vaccinated trial population showed that RSVPreF3 OA was well tolerated and had an acceptable safety profile; most solicited adverse events were transient with mild to moderate severity, and no imbalances were observed in the rates of serious adverse events or potential immune-mediated diseases [27]. Together, these results indicate a positive benefit-risk balance of the vaccine in older adults, including those with coexisting medical conditions.

The analyses presented here have some limitations. The efficacy and immunogenicity analyses in the different subgroups were descriptive and were not adjusted for multiplicity. However, the magnitude of the vaccine efficacy estimates and high CI lower limits support the statistical robustness of these analyses. Although the proportion of older adults with coexisting medical conditions and the type of conditions were as expected from this population of older adults [22, 30, 31], enrollment was restricted to those who were considered medically stable. However, efficacy against RSV-LRTD and RSV-ARI remained high, with an increasing number of underlying conditions (92.0% and 88.0%, respectively, among older adults with at least 2 medical conditions of interest), suggesting that the vaccine may protect some of the most vulnerable older adults with multimorbidity.

In conclusion, this phase 3 trial showed that a single dose of the adjuvanted RSVPreF3 OA vaccine was immunogenic and efficacious in preventing RSV-LRTD and RSV-ARI in older adults with cardiorespiratory and endocrine or metabolic conditions associated with an increased risk of severe RSV disease.

## Supplementary Data

Supplementary materials are available at *Clinical Infectious Diseases* online. Consisting of data provided by the authors to benefit the reader, the posted materials are not copyedited and are the sole responsibility of the authors, so questions or comments should be addressed to the corresponding author.

## Supplementary Material

ciad471_Supplementary_Data

## Notes

***Author contributions*.** L. F., M. P. D., C. M., M. V. d. W., L. K., and V. H. were involved in the conception or design of the study/project. R. G. F., R. A. I., K. S., D. G. L., A. P., and M. G. I. participated in the acquisition or generation of the data. L. F., M. P. D., C. M., L. K., and V. H. performed the data analysis. K. S., L. F., M. P. D., C. M., L. K., and V. H. were involved in the interpretation of the data.

***Acknowledgments*.** The authors thank all the participants, study team members, and site staff for their contributions. Support for medical writing (by Natalie Denef), design, and manuscript coordination were provided by Akkodis Belgium (c/o GSK). The AReSVi-006 Study Group members are (listed alphabetically): Adams, Mark; Adams, Michael; Akite, Elaine Jacqueline; Alt, Ingrid; Andrews, Charles; Asatryan, Asmik; Athan, Eugene; Bahrami, Ghazaleh; Bargagli, Elena; Bhorat, Qasim; Bird, Paul; Borowy, Przemyslaw; Boutry, Celine; Brotons Cuixart, Carles; Browder, David; Brown, Judith; Buntinx, Erik; Cameron, Donald; Campora, Laura; Chinsky, Kenneth; Choi, Melissa; Choo, Eun-Ju; Collete, Delphine; Corral Carrillo, Maria; Davis, Matthew G; de Heusch, Magali; de Looze, Ferdinandus; De Meulemeester, Marc; De Negri, Ferdinando; De Schrevel, Nathalie; DeAtkine, David; Dedkova, Viktoriya; Descamps, Dominique; Dezutter, Nancy; Dzongowski, Peter; Eckermann, Tamara; Essink, Brandon; Faulkner, Karen; Ferguson, Murdo; Fuller, Gregory; Galan Melendez, Isabel Maria; Gentile, Ivan; Ghesquiere, Wayne; Grimard, Doria; Gruselle, Olivier; Halperin, Scott; Heer, Amardeep; Helman, Laura; Hotermans, Andre; Jelinek, Tomas; Kamerbeek, Jackie; Kim, Hyo Youl; Kimmel, Murray; Koch, Mark; Kokko, Satu; Koski, Susanna; Kotb, Shady; Lalueza, Antonio; Langley, Joanne M; Lee, Jin-Soo; Leroux-Roels, Isabel; Lins, Muriel; Lombaard, Johannes; Mahomed, Akbar; Malerba, Mario; Marechal, Celine; Martinon-Torres, Federico; Martinot, Jean-Benoit; Masuet-Aumatell, Cristina; McNally, Damien; Medina Pech, Carlos Eduardo; Mendez Galvan, Jorge; Mesaros, Narcisa Elena; Mesotten, Dieter; Mitha, Essack; Mngadi, Kathryn; Moeckesch, Beate; Montgomery, Barnaby; Murray, Linda; Nally, Rhiannon; Narejos Perez, Silvia; Newberg, Joseph; Nugent, Paul; Ochoa Mazarro, Dolores; Oda, Harunori; Olivier, Aurelie; Orso, Maurizio; Ortiz Molina, Jacinto; Pak, Tatiana; Park, Dae Won; Patel, Meenakshi; Patel, Minesh; Pedro Pijoan, Anna Maria; Perez Vera, Merce; Perez, Alberto Borobia; Perez-Breva, Lina; Pileggi, Claudia; Pregliasco, Fabrizio; Pretswell, Carol; Quinn, Dean; Reynolds, Michele; Romanenko, Viktor; Rosen, Jeffrey; Roy, Nathalie; Ruiz Antoran, Belen; Sakata, Hideaki; Sauter, Joachim; Schaefer, Axel; Schwarz, Tino F; Sein Anand, Izabela; Serra Rexach, Jose Antonio; Shu, David; Siig, Andres; Simon, William; Smakotina, Svetlana; Stephan, Brigitte; Tafuri, Silvio; Takazawa, Kenji; Tellier, Guy; Terryn, Wim; Tharenos, Leslie; Thomas, Nick; Toursarkissian, Nicole; Ukkonen, Benita; Vale, Noah; Van Landegem, Pieter-Jan; van Zyl-Smit, Richard N; Vanden Abeele, Carline; Verheust, Celine; Vermeersch, Lode; Vicco, Miguel; Vitale, Francesco; Voloshyna, Olga; White, Judith; Wie, Seong-Heon; Wilson, Jonathan; Ylisastigui, Pedro.

***Data availability*.** Anonymized data can be requested from www.clinicalstudydatarequest.com after completion of the trial.

***Trademark statement*.** AS01 and Arexvy are trademarks owned by or licensed to GSK.

***Financial support*.** This work was supported by GlaxoSmithKline Biologicals SA, who funded this trial, was involved in all stages of trial conduct, and took charge of all costs associated with the development and publication of this manuscript.
