# Supplementary material for: Respiratory Syncytial Virus Prefusion F Protein Vaccine Is Efficacious in Older Adults With Underlying Medical Conditions
Source: Clin Infect Dis. 2023 Sep 12;78(1):202–9. doi: 10.1093/cid/ciad471 (PMC10810713; doi:10.1093/cid/ciad471)
Supplement: ciad471_Supplementary_Data [file ciad471_supplementary_data.docx]

**Supplementary material**

**Supplementary methods**

***Case definition for acute respiratory illness (ARI)***

Presence of:

- at least two respiratory symptoms/signs for at least 24 hours

OR

- at least one respiratory symptom/sign + one systemic symptom/sign for at least 24 hours

Respiratory symptoms/signs were:

- Nasal congestion/rhinorrhea
- Sore throat
- New or increased sputum
- New or increased cough
- New or increased dyspnea (shortness of breath)
- New or increased wheezing (reported by the participant or investigator)
- New or increased crackles/ronchi (reported by the investigator) based on chest auscultation
- Respiratory rate ≥20 respirations/min (reported by the investigator)
- Low or decreased oxygen saturation (= oxygen saturation <95% or ≤90% if pre-season baseline was <95%, reported by the investigator)
- Need for oxygen supplementation (reported by the investigator)

Systemic symptoms/signs were:

- Fever (body temperature ≥38.0°C/100.4°F by any route) or feverishness (feeling of having fever without objective measurement)
- Fatigue
- Body aches
- Headache
- Decreased appetite

***Case definition for lower respiratory tract disease (LRTD)***

Presence of:

- at least two lower respiratory symptoms/signs for at least 24 hours including at least one lower respiratory sign

OR

- at least three lower respiratory symptoms for at least 24 hours

Lower respiratory symptoms were:

- New or increased sputum
- New or increased cough
- New or increased dyspnea (shortness of breath)

Lower respiratory signs were:

- New or increased wheezing (reported by the participant or investigator)
- New or increased crackles/ronchi (reported by the investigator) based on chest auscultation
- Respiratory rate ≥20 respirations/min (reported by the investigator)
- Low or decreased oxygen saturation (= oxygen saturation <95% or ≤90% if pre-season baseline was <95%, reported by the investigator)
- Need for oxygen supplementation (reported by the investigator)

***ARI and LRTD review and adjudication process***

- For each ARI, the investigators had to record information on respiratory signs and symptoms and systemic symptoms in the electronic case report form (eCRF). In addition, they had to provide a clinical diagnosis and assess if the ARI was considered as an LRTD based on their medical judgment.
- All ARI cases reported by the investigators as meeting the ARI case definition were reviewed by a case coordinator to make sure the recorded data were complete and accurate. Subsequently, a case validator reviewed the ARI cases identified by the case coordinator to determine whether these ARIs met the case definitions of LRTD or severe LRTD based on the protocol-defined list of signs and symptoms. At the end of this review process, all ARI cases fulfilling the protocol-defined LRTD case definition and all ARI cases that were clinically diagnosed as LRTD by the investigators but did not strictly meet the case definition were marked as “ready for adjudication”. These cases were matched with the respiratory syncytial virus (RSV) reverse transcriptase-polymerase chain reaction (RT-PCR) results, and all cases marked as “ready for adjudication” that had a positive RSV RT-PCR result were sent to the external adjudication committee.
- Two adjudication committee reviewers independently evaluated these cases based on the adjudication information package they received (which contained a subset of eCRF data related to demography, general medical history, ARI data [vital signs recorded during the ARI visit, ARI symptoms, ARI complications], unsolicited adverse events, medications, smoking status, and COVID-19 data). Each adjudication committee reviewer documented their decision on whether they adjudicated the case as an LRTD or severe LRTD based on the case definitions. If their decisions did not match, the committee chairperson evaluated the case and determined the final adjudication (if needed through an alignment meeting with the reviewers).
- Only the adjudicated cases were included in the analyses presented in the current manuscript.

**Supplementary results**

***Vaccine efficacy against RSV disease with medically attended visits***

Of the 47 participants with an adjudicated RSV-LRTD, 27 (57.4%) required medically attended visits. Of these, 3 were in the RSVPreF3 OA group (incidence: 0.4/1000 person-years) and 24 in the placebo group (incidence: 3.5/1000 person-years), resulting in a vaccine efficacy of 87.5% (95% confidence interval [CI]: 58.9–97.6) against RSV-LRTD with medically attended visits.

Of the 122 participants with an RSV-ARI, 46 (37.7%) required medically attended visits. Of these, 8 were in the RSVPreF3 OA group (incidence: 1.2/1000 person-years) and 38 in the placebo group (incidence: 5.5/1000 person-years), resulting in a vaccine efficacy of 79.0% (95% CI: 54.3–91.5) against RSV-ARI with medically attended visits.

**Supplementary tables**

**Supplementary Table 1. Most common co-existing medical conditions by Medical Dictionary for Regulatory Activities Terminology system organ class and high-level term (exposed population)**

| **System organ class** | **RSVPreF3 OA**  **N=12,467** | | **Placebo**  **N=12,499** | | **Total**  **N=24,966** | |
| --- | --- | --- | --- | --- | --- | --- |
| High-level term | **n** | **%** | **n** | **%** | **n** | **%** |
| **Any co-existing condition** | 11,929 | 95.7 | 11,905 | 95.2 | 23,834 | 95.5 |
| **Vascular disorders** | 7551 | 60.6 | 7440 | 59.5 | 14,991 | 60.0 |
| Vascular hypertensive disorders NEC | 7208 | 57.8 | 7085 | 56.7 | 14,293 | 57.2 |
| Peripheral vascular disorders NEC | 419 | 3.4 | 416 | 3.3 | 835 | 3.3 |
| Varicose veins NEC | 313 | 2.5 | 295 | 2.4 | 608 | 2.4 |
| **Metabolism and nutrition disorders** | 7247 | 58.1 | 7350 | 58.8 | 14,597 | 58.5 |
| Elevated cholesterol | 3138 | 25.2 | 3057 | 24.5 | 6195 | 24.8 |
| Diabetes mellitus (including subtypes) | 2839 | 22.8 | 2885 | 23.1 | 5724 | 22.9 |
| Hyperlipidemias NEC | 1810 | 14.5 | 1845 | 14.8 | 3655 | 14.6 |
| General nutritional disorders NEC | 1096 | 8.8 | 1123 | 9.0 | 2219 | 8.9 |
| Lipid metabolism and deposit disorders NEC | 1069 | 8.6 | 1112 | 8.9 | 2181 | 8.7 |
| Disorders of purine metabolism | 702 | 5.6 | 718 | 5.7 | 1420 | 5.7 |
| Fat soluble vitamin deficiencies and disorders | 239 | 1.9 | 260 | 2.1 | 499 | 2.0 |
| **Musculoskeletal and connective tissue disorders** | 6515 | 52.3 | 6478 | 51.8 | 12,993 | 52.0 |
| Osteoarthropathies | 4214 | 33.8 | 4159 | 33.3 | 8373 | 33.5 |
| Musculoskeletal and connective tissue pain and discomfort | 2220 | 17.8 | 2267 | 18.1 | 4487 | 18.0 |
| Metabolic bone disorders | 970 | 7.8 | 986 | 7.9 | 1956 | 7.8 |
| Intervertebral disc disorders NEC | 412 | 3.3 | 448 | 3.6 | 860 | 3.4 |
| Joint-related signs and symptoms | 403 | 3.2 | 362 | 2.9 | 765 | 3.1 |
| Spine and neck deformities | 264 | 2.1 | 272 | 2.2 | 536 | 2.1 |
| Joint-related disorders NEC | 256 | 2.1 | 244 | 2.0 | 500 | 2.0 |
| **Gastrointestinal disorders** | 4285 | 34.4 | 4144 | 33.2 | 8429 | 33.8 |
| Gastrointestinal atonic and hypomotility disorders NEC | 2852 | 22.9 | 2824 | 22.6 | 5676 | 22.7 |
| Peptic ulcers and perforation | 311 | 2.5 | 284 | 2.3 | 595 | 2.4 |
| Diverticula | 267 | 2.1 | 259 | 2.1 | 526 | 2.1 |
| Gastritis (excluding infective) | 256 | 2.1 | 251 | 2.0 | 507 | 2.0 |
| **Respiratory, thoracic, and mediastinal disorders** | 3868 | 31.0 | 3821 | 30.6 | 7689 | 30.8 |
| Bronchospasm and obstruction | 2165 | 17.4 | 2092 | 16.7 | 4257 | 17.1 |
| Breathing abnormalities | 1101 | 8.8 | 1092 | 8.7 | 2193 | 8.8 |
| Nasal congestion and inflammations | 906 | 7.3 | 944 | 7.6 | 1850 | 7.4 |
| **Surgical and medical procedures** | 3828 | 30.7 | 3826 | 30.6 | 7654 | 30.7 |
| Joint therapeutic procedures | 931 | 7.5 | 942 | 7.5 | 1873 | 7.5 |
| Uterine therapeutic procedures | 682 | 5.5 | 702 | 5.6 | 1384 | 5.5 |
| Biliary tract and gallbladder therapeutic procedures | 532 | 4.3 | 522 | 4.2 | 1054 | 4.2 |
| Large intestine therapeutic procedures | 498 | 4.0 | 488 | 3.9 | 986 | 3.9 |
| Lens therapeutic procedures | 471 | 3.8 | 476 | 3.8 | 947 | 3.8 |
| Arterial therapeutic procedures (excluding aortic) | 378 | 3.0 | 370 | 3.0 | 748 | 3.0 |
| Tonsillar therapeutic procedures | 297 | 2.4 | 289 | 2.3 | 586 | 2.3 |
| Spine and spinal cord therapeutic procedures | 277 | 2.2 | 291 | 2.3 | 568 | 2.3 |
| Hernia repairs | 258 | 2.1 | 271 | 2.2 | 529 | 2.1 |
| Therapeutic procedures NEC | 198 | 1.6 | 253 | 2.0 | 451 | 1.8 |
| **Eye disorders** | 3556 | 28.5 | 3510 | 28.1 | 7066 | 28.3 |
| Cataract conditions | 2396 | 19.2 | 2390 | 19.1 | 4786 | 19.2 |
| Refractive and accommodative disorders | 657 | 5.3 | 630 | 5.0 | 1287 | 5.2 |
| Glaucomas (excluding congenital) | 455 | 3.6 | 471 | 3.8 | 926 | 3.7 |
| **Psychiatric disorders** | 3076 | 24.7 | 3180 | 25.4 | 6256 | 25.1 |
| Depressive disorders | 1623 | 13.0 | 1702 | 13.6 | 3325 | 13.3 |
| Disturbances in initiating and maintaining sleep | 1286 | 10.3 | 1318 | 10.5 | 2604 | 10.4 |
| Anxiety symptoms | 1051 | 8.4 | 1080 | 8.6 | 2131 | 8.5 |
| **Immune system disorders** | 2925 | 23.5 | 2870 | 23.0 | 5795 | 23.2 |
| Atopic disorders | 1947 | 15.6 | 1986 | 15.9 | 3933 | 15.8 |
| Allergies to foods, food additives, drugs, and other chemicals | 1290 | 10.3 | 1237 | 9.9 | 2527 | 10.1 |
| **Nervous system disorders** | 2614 | 21.0 | 2612 | 20.9 | 5226 | 20.9 |
| Central nervous system hemorrhages and cerebrovascular accidents | 379 | 3.0 | 365 | 2.9 | 744 | 3.0 |
| Migraine headaches | 335 | 2.7 | 369 | 3.0 | 704 | 2.8 |
| Headaches NEC | 316 | 2.5 | 311 | 2.5 | 627 | 2.5 |
| Peripheral neuropathies NEC | 299 | 2.4 | 319 | 2.6 | 618 | 2.5 |
| Sensory abnormalities NEC | 257 | 2.1 | 299 | 2.4 | 556 | 2.2 |
| **Cardiac disorders** | 2582 | 20.7 | 2600 | 20.8 | 5182 | 20.8 |
| Ischemic coronary artery disorders | 1000 | 8.0 | 999 | 8.0 | 1999 | 8.0 |
| Coronary artery disorders NEC | 853 | 6.8 | 888 | 7.1 | 1741 | 7.0 |
| Supraventricular arrhythmias | 753 | 6.0 | 759 | 6.1 | 1512 | 6.1 |
| Heart failures NEC | 421 | 3.4 | 422 | 3.4 | 843 | 3.4 |
| **Reproductive system and breast disorders** | 2065 | 16.6 | 2085 | 16.7 | 4150 | 16.6 |
| Prostatic neoplasms and hypertrophy | 1336 | 10.7 | 1378 | 11.0 | 2714 | 10.9 |
| Erection and ejaculation conditions and disorders | 275 | 2.2 | 280 | 2.2 | 555 | 2.2 |
| **Neoplasms benign, malignant, and unspecified (including cysts and polyps)** | 2014 | 16.2 | 1989 | 15.9 | 4003 | 16.0 |
| Skin neoplasms malignant and unspecified (excluding melanoma) | 529 | 4.2 | 576 | 4.6 | 1105 | 4.4 |
| Breast and nipple neoplasms malignant | 290 | 2.3 | 303 | 2.4 | 593 | 2.4 |
| Urinary tract neoplasms malignant NEC | 310 | 2.5 | 277 | 2.2 | 587 | 2.4 |
| **Renal and urinary disorders** | 1979 | 15.9 | 1943 | 15.5 | 3922 | 15.7 |
| Bladder and urethral symptoms | 646 | 5.2 | 682 | 5.5 | 1328 | 5.3 |
| Renal failure and impairment | 444 | 3.6 | 442 | 3.5 | 886 | 3.5 |
| Renal lithiasis | 402 | 3.2 | 404 | 3.2 | 806 | 3.2 |
| Myoneurogenic bladder disorders | 307 | 2.5 | 313 | 2.5 | 620 | 2.5 |
| **Infections and infestations** | 1911 | 15.3 | 1805 | 14.4 | 3716 | 14.9 |
| Upper respiratory tract infections | 336 | 2.7 | 326 | 2.6 | 662 | 2.7 |
| Abdominal and gastrointestinal infections | 332 | 2.7 | 319 | 2.6 | 651 | 2.6 |
| Herpes viral infections | 289 | 2.3 | 308 | 2.5 | 597 | 2.4 |
| **Endocrine disorders** | 1805 | 14.5 | 1894 | 15.2 | 3699 | 14.8 |
| Thyroid hypofunction disorders | 1447 | 11.6 | 1533 | 12.3 | 2980 | 11.9 |
| Thyroid disorders NEC | 248 | 2.0 | 256 | 2.0 | 504 | 2.0 |
| **Injury, poisoning, and procedural complications** | 1312 | 10.5 | 1220 | 9.8 | 2532 | 10.1 |
| Limb fractures and dislocations | 631 | 5.1 | 574 | 4.6 | 1205 | 4.8 |
| **Skin and subcutaneous tissue disorders** | 1147 | 9.2 | 1172 | 9.4 | 2319 | 9.3 |
| Dermatitis and eczema | 436 | 3.5 | 400 | 3.2 | 836 | 3.3 |
| **Hepatobiliary disorders** | 959 | 7.7 | 987 | 7.9 | 1946 | 7.8 |
| Cholecystitis and cholelithiasis | 466 | 3.7 | 433 | 3.5 | 899 | 3.6 |
| Hepatocellular damage and hepatitis NEC | 444 | 3.6 | 454 | 3.6 | 898 | 3.6 |
| **Ear and labyrinth disorders** | 792 | 6.4 | 796 | 6.4 | 1588 | 6.4 |
| Hearing losses | 456 | 3.7 | 445 | 3.6 | 901 | 3.6 |
| Inner ear signs and symptoms | 281 | 2.3 | 330 | 2.6 | 611 | 2.4 |
| **Social circumstances** | 669 | 5.4 | 695 | 5.6 | 1364 | 5.5 |
| Age-related issues | 546 | 4.4 | 557 | 4.5 | 1103 | 4.4 |
| **Investigations (e.g., imaging and laboratory analyses)** | 576 | 4.6 | 590 | 4.7 | 1166 | 4.7 |
| **General disorders and administration site conditions** | 531 | 4.3 | 479 | 3.8 | 1010 | 4.0 |
| **Blood and lymphatic system disorders** | 310 | 2.5 | 317 | 2.5 | 627 | 2.5 |

Table shows system organ classes and high-level terms for which co-existing conditions were reported for ≥2.0% of participants in either of the groups.

RSVPreF3 OA, group with participants who received a single dose of AS01_E_-adjuvanted respiratory syncytial virus (RSV) prefusion F protein-based vaccine; placebo, group with participants who received a single dose of placebo; N, number of participants in the exposed population; n/%, number/percentage of participants reporting the indicated condition; NEC, not elsewhere classified.

**Supplementary Table 2. RSV-A and RSV-B neutralizing titers and RSVPreF3-binding IgG concentrations** **before and 1 month after RSVPreF3 OA or placebo administration, by co-existing medical conditions of interest (per-protocol population for immunogenicity)**

| **Subgroup** | **Timepoint** | **RSVPreF3 OA** | | | |  | **Placebo** | | | | |
| --- | --- | --- | --- | --- | --- | --- | --- | --- | --- | --- | --- |
|  |  | **N** | **GMT, ED60 or GMC, EU/mL (95% CI)** | **N’** | **GMI (95% CI)** |  | **N** | **GMT, ED60 or GMC, EU/mL (95% CI)** | **N’** | **GMI (95% CI)** | |
| **RSV-A neutralizing titers** | | | | | | | | | | |  |
| No condition of interest | Day 1 | 557 | 885.4 (822.7–952.9) |  |  |  | 581 | 886.4 (826.7–950.5) |  |  | |
|  | Day 31 | 532 | 8670.5 (7949.7–9456.7) | 530 | 9.8 (8.9–10.6) |  | 553 | 844.6 (782.5–911.6) | 553 | 1.0 (0.9–1.0) | |
| ≥1 condition of interest | Day 1 | 328 | 976.1 (885.4–1076.2) |  |  |  | 311 | 1012.7 (919.9–1114.9) |  |  | |
|  | Day 31 | 316 | 10,554.7 (9387.1–11,867.6) | 314 | 11.0 (9.6–12.5) |  | 293 | 931.0 (844.7–1026.2) | 293 | 0.9 (0.9–1.0) | |
| **RSV-B neutralizing titers** | | | | | | | | | | |  |
| No condition of interest | Day 1 | 557 | 1132.0 (1056.1–1213.3) |  |  |  | 581 | 1189.4 (1108.8–1275.9) |  |  | |
|  | Day 31 | 532 | 9320.3 (8617.0–10,081.0) | 530 | 8.3 (7.7–9.0) |  | 553 | 1203.7 (1113.4–1301.3) | 553 | 1.0 (1.0–1.1) | |
| ≥1 condition of interest | Day 1 | 328 | 1312.5 (1193.8–1443.0) |  |  |  | 311 | 1353.1 (1223.6–1496.3) |  |  | |
|  | Day 31 | 316 | 11,806.7 (10,673.7–13,060.1) | 314 | 9.1 (8.1–10.2) |  | 293 | 1383.3 (1238.5–1545.1) | 293 | 1.0 (1.0–1.1) | |
| **RSVPreF3-binding IgG concentrations** | | | | | | | | | | |  |
| No condition of interest | Day 1 | 557 | 6621.5 (6249.9–7015.3) |  |  |  | 581 | 6891.1 (6530.9–7271.2) |  |  | |
|  | Day 31 | 532 | 85,599.4 (80,799.3–90,684.8) | 530 | 12.9 (12.1–13.9) |  | 553 | 6839.4 (6458.2–7243.1) | 553 | 1.0 (1.0–1.0) | |
| ≥1 condition of interest | Day 1 | 328 | 7815.3 (7226.8–8451.7) |  |  |  | 311 | 7477.2 (6929.4–8068.4) |  |  | |
|  | Day 31 | 316 | 103,058.7 (95,193.9–111,573.3) | 314 | 13.3 (12.0–14.8) |  | 293 | 7448.3 (6889.5–8052.4) | 293 | 1.0 (1.0–1.0) | |

Respiratory syncytial virus (RSV) subtypes A and B neutralizing titers and RSVPreF3-binding immunoglobulin G (IgG) concentrations, measured by enzyme-linked immunosorbent assay on samples collected before (day 1) and 1 month after (day 31) administration of the AS01_E_-adjuvanted RSV prefusion F protein-based vaccine (RSVPreF3 OA) or placebo for the subgroups of participants without any of the co-existing medical conditions of interest (no condition of interest) or with at least one of these conditions (≥1 condition of interest); conditions of interest were cardiorespiratory conditions (any chronic respiratory or pulmonary disease [including chronic obstructive pulmonary disease, asthma, and other conditions], chronic heart failure) and endocrine and metabolic conditions (diabetes mellitus type 1 or type 2 and advanced liver or renal disease) that are associated with an increased risk of severe RSV disease.

N, number of participants with available results at the indicated timepoint; N’, number of participants with available results at both timepoints; GMT, geometric mean titer; ED60, estimated dilution 60; GMC, geometric mean concentration; EU, enzyme-linked immunosorbent assay units; CI, confidence interval; GMI, geometric mean increase in titer or concentration from day 1 to day 31.
